# The convergence of American and Nigerian religious conservatism in a biopolitical shaping of Nigeria's HIV/AIDS prevention programmes

**DOI:** 10.1080/17441692.2013.765023

**Published:** 2013-02-08

**Authors:** Jlateh V. Jappah

**Affiliations:** Institute for Global Health, University College London, London, UK

**Keywords:** Nigeria, religious conservatism, PEPFAR, HIV/AIDS, biopolitics

## Abstract

Nigeria has the largest number of HIV/AIDS cases in West Africa, with 3.3 million people estimated to be living with the disease. The country remains a fragile democratic state and has allocated insufficient resources to combat the spread of HIV/AIDS among its citizens. The preponderance of President's Emergency Plan for AIDS Relief (PEPFAR) dollars, expert knowledge, conservative ideology and activities has shaped the direction of HIV/AIDS sexual-transmission prevention programmes in Nigeria. PEPFAR channels significant resources through Nigerian faith-based organisations (FBOs), and considers these organisations integral for HIV prevention strategies. In many instances, HIV/AIDS prevention programmes managed by FBOs reflect their ideologies of morality and sexuality. There is a convergence of religious ideology concerning morality and HIV infectivity between American and Nigerian conservatives; this produces a fertile ground for the influence and expansion of the conservative activities of PEPFAR in Nigeria. The paper highlights this nexus and draws attention to the biopolitical underpinning of PEPFAR in shaping Nigeria's HIV prevention programmes. The paper further notes both positive and negative effects of PEPFAR activities and attempts by the Obama administration to redirect PEPFAR to a more holistic approach in order to optimise outcomes.

## Introduction

Within the past two decades, African states have been transitioning from autocratic rule to more representative forms of governance. Bassett and Straus (2011) note that in 1989, only five states in sub-Saharan Africa had democratic multiparty political systems. Conversely, they note that by the late 1990s, the trend had changed with only four countries in the region that had not transitioned to holding multiparty presidential or parliamentary elections. However, Bassett and Straus attest that the rise of democratic governance in sub-Saharan Africa has not been flawless, and cite instances of fraudulent elections and coup d’états. Yet this new trend of representation has been spreading and has spurred a new wave of civil societies and grassroots organisations demanding rights and entitlement programmes from their governments and the international community at large. Further, De Waal (2006) notes that African electorates have not made HIV/AIDS a top priority despite the havoc the disease continues to wreak. He argues that despite the conspicuous inaction or denialism exhibited by many African leaders regarding HIV/AIDS, the disease does not threaten the political power of the continent's leaders. Against this background, De Waal argues that African leaders’ responses to HIV/AIDS lack the urgency and scale commensurate with its devastation.

Nigeria is no exception in following a similar trend. The country has the largest number of HIV/AIDS cases in West Africa. The burden of HIV/AIDS has increased significantly; 3.3 million people are estimated to be living with the disease today in Nigeria, and it ranks third in the world in terms of absolute numbers, after South Africa and India. Also, 220,000 deaths occur annually from AIDS in Nigeria, ranking it second globally (UNAIDS 2010). The inadequate attention given to HIV/AIDS health-related matters is partly caused by a lack of political stability in Nigeria over the years. The country has endured a history of successive military regimes and currently has a fragile democratic government and an increasing radical Islamic militancy. Sufficient resources have not been allocated by the government to combat the spread of HIV/AIDS. Nigeria's HIV policies have been mainly donor driven, and the US President's Emergency Plan for AIDS Relief (PEPFAR) plays the lead role.

The USA recognises Nigeria as a strategic partner in the region, both for its large Muslim population and its hydrocarbon wealth (Ingram 2007; Carson 2010). Johnnie Carson, the US Assistant Secretary of State, provides critical insight into US policy in Nigeria when he states that ‘Nigeria is one of the two most important countries in sub-Saharan Africa, and what happens in Nigeria has consequences for Africa, the United States, and the global community’ (Carson 2011, p. 4). Nigeria's peacekeeping efforts have been unparalleled in maintaining the security of the region, especially in former trouble spots like Liberia, Sierra Leone and Cộte d'Ivoire. HIV prevalence among Nigerian uniformed personnel has been an important policy subject. It has been suggested that military personnel serve as vectors for HIV and have the potential to foster the spread of HIV/AIDS in neighbouring countries. The USA has allocated a large amount of money to Nigeria to combat the spread of the disease.

The US government has been pivotal in formulating health policies geared toward improving the health status of Nigerians. PEPFAR HIV/AIDS activities include antiretroviral treatment, prevention of HIV infection from mother to child and HIV/AIDS awareness programmes for military personnel and the general public. PEPFAR is also involved in improving the country's health care infrastructure and institutional capacity. A strategic aim of PEPFAR is to change sexual practices and behaviours. PEPFAR collaborates with Nigerian government agencies, local Nigerian and international non-governmental and faith-based organisations (FBOs), as well as US government agencies and universities to implement HIV/AIDS relief programmes. FBOs are prominent PEPFAR implementation partners. Among them are Catholic Relief Services AIDS Relief, Christian Health Association of Nigeria, Christian Aid Community Based Care of Orphans and Vulnerable Children (OVC), Christian AID Community Care Nigeria, Catholic Relief Services 7 Diocese, Catholic Relief Services – Scale Up Nigeria OVC and Catholic Secretariat of Nigeria (United States Diplomatic Mission to Nigeria 2012). As PEPFAR administrators, US bureaucrats and technical experts have been highly influential in formulating Nigerian HIV/AIDS prevention policies and in framing the Nigerian National Strategic Framework (2010), the country's HIV/AIDS policy document. In this paper, I highlight a shared conservative ideology between Nigerian and American religious/political leaders. This ideology underpins the foundation of HIV/AIDS sexual-transmission prevention programmes in Nigeria through a biopolitical production of life.

## The biopolitical conceptual framework

Foucault (1998) writes that since the Classical Age, Western societies have transitioned from means of deduction (taking of life) to exert a positive influence on life (production of life). He notes that ‘… the ancient right to take life or let live was replaced by a power to foster life or disallow it to the point of death’ (p. 138). According to Foucault:

Power would no longer be dealing simply with legal subjects over whom the ultimate dominion was death, but with living beings, and the mastery it would be able to exercise over them would have to be applied at the level of life itself; it was the taking charge of life, more than the threat of death, that gave power its access even to the body. (pp. 142–143)

This paradigm seeks to harness life, as the influence of power is only applied to living beings, and death represents the limits of power. Political power now predominantly regards man through the lens of his biological characteristics, characteristics that are intertwined with his economic potential within a collective body. Its highest function is to invest in the production of a healthy life, which will ensure the economic functions of the body.

Foucault notes that governmental management in the biopolitical economy of power aims to regulate/maximise the population's health by averting diseases and accidents, lowering mortality rates, increasing longevity, stimulating fertility and birth rates, increasing economic productivity and so on. It involves the governing of population health, with statistical applications categorising diseases and non-diseases, as well as the prevalence and incidence of diseases, trends, projections and consequences of diseases if left untreated. As such, Elbe (2005, p. 406) contends that ‘… disease could no longer be left to the random fluctuations of nature, but would have to be brought under continuous political and social control’.

Governments engage in shaping demographic indicators for the betterment of populations. Foucault writes that ‘for capitalist society, it is the biopolitical which was of first importance, the biological, the somatic, the bodily. The body is above all a biopolitical reality; medicine is a biopolitical strategy’ (quoted in Curtis 2002, p. 512). According to Foucault, the biopolitical form of power focuses on ‘man as a species’ and is aimed at the collective body (population). It forms the basis of the biological process with the aim of increasing population productivity through control and governmental intervention. Thus, biopolitics is a subsection of politics (polis) that focuses on the biological life/human population (bio).

Foucault (1998) observes:

Power must be analyzed as something, which circulates, or rather something, which only functions in the form of a chain. It is never localised here or there, never in anybody's hands, never appropriated as a commodity or piece [of] wealth. Power is employed and exercised through a netlike organization. (p. 98)

The confluence of power extends beyond territorial boundaries. As such, Mbembe (2003) notes that ‘it makes little sense to insist on distinctions between “internal” and “external” political realms, separated by clearly demarcated boundaries’ (pp. 31–32). In an attempt to protect population health, powers in the West appear to be moving from the competitive nature of Westphalian nation-states to a model that galvanises sovereign entities into prioritising the welfare of populations.

## The biopolitics of PEPFAR: Nigeria

Nigeria plays a strategic role in the region. It is Africa's second-largest economy and the most populous country in Africa. In order to avert a fate similar to that of many Southern African countries in which approximately one-third of the adult population is HIV positive, it is imperative to address the HIV/AIDS epidemic in Nigeria. At the crucial juncture of global HIV unease, the US government took the lead.

Addressing the USA Congress in 2003, President George W. Bush asserted that ‘… seldom has history afforded a greater opportunity to do so much for so many’ (PEPFAR 2011), when announcing the USA's plan to respond to the global HIV/AIDS pandemic. He emphasised the need for the USA to seize the opportunity in the time of HIV/AIDS unease to act compassionately in combating the disease. PEPFAR became the masterpiece of the president's vision. PEPFAR was approved by the USA Congress in May 2003. It allocated US$15 billion to combat the spread of HIV/AIDS, tuberculosis and malaria globally within a five-year period beginning in 2004 to the end of 2008. The ambitious plan aimed to provide a comprehensive strategy to prevent 7 million new HIV cases, treat at least 2 million people with life-extending HIV/AIDS drugs and provide human care for 10 million people with the disease, including children who became orphans due to AIDS. A Reauthorization Act was signed into law in 2008, just before President Bush left office, allocating an additional US$48 billion for the years 2009 through 2013 for PEPFAR activities. PEPFAR has been cited as either the only or the most successful story of the Bush Administration.

The politics of PEPFAR have been and continue to be highly polemical, with different interest groups contesting the direction of PEPFAR, especially prevention programmes that address the sexual transmission of HIV. Evangelical Christians in the USA and their counterparts in Africa, the Catholic Church and other FBOs, gender-based advocacy groups, human rights organisations, social movements, health experts and politicians have vied to tilt the pendulum of PEPFAR in their direction. American national politics of social conservatism versus progressivism over morality and sexuality have been brought to the global stage, engaging both supporters and dissenters.

Religious conservative ideology played an important role in the formulation of PEPFAR (Ingram 2007, Leventhal 2010, Pereira 2011). Evertz (2010) traces PEPFAR's ideological stance from the Reagan Republican Administration through the passage of the Adolescent Family Life Act in 1981, which aimed to prevent premarital teen pregnancy in the USA by promoting chastity and self-discipline. He notes that earlier government grants for the programme were given ‘exclusively to far-right and religious groups. … developing programs that explicitly promoted religious values’ (p. 7).

In formulating PEPFAR legislation, President Bush, a born-again Christian, responded to his religious base. Ingram (2005) argues that the implementation of global AIDS relief by the Bush Administration was partly in response to pressure from evangelical Christian groups in the USA. These groups shaped the legislation to reflect their core conservative values. Ingram (2007) highlights that ‘the moralizing dimension of US HIV/AIDS relief carries strong echoes of colonial and missionary projects of civilization, but encounters African societies themselves undergoing religious revivalism’ (p. 527). Pereira (2011, p. 2) writes in a similar vein that ‘biomedicine in the world's former colonial territories was introduced as a tool of civilization and evangelization by Christian missions which pioneered and largely dominated the field—as well as by philanthropic entities and states authorities’. Senate Majority Leader Bill Frist and Senator Jesse Helms (who had earlier claimed that HIV/AIDS mainly affected people who engaged in sodomy), played a crucial role as conservatives in the formulation of PEPFAR (Dietrich 2007). Bush and fellow conservatives became passionate about helping HIV victims based on their belief in their moral responsibility to assist the sick and help restore life.

Phyllis Schlafly (2003), an influential conservative activist, wrote President Bush a month before PEPFAR was signed into law, urging the president to sign a bill only if it contained legislative language codifying his priorities. She suggested that such priorities include changing behaviour, abstinence, faithfulness and avoidance of abortion. This born-again doctrine was argued to be the most effective and low-cost way to mitigate the spread of HIV. Schlafly implored the president to make ‘A’ (abstain) and ‘B’ (be faithful) leading priorities in PEPFAR legislation. Schlafly advocated that PEPFAR prevention funds be based on what she claimed to be ‘Uganda's “ABC” approach’ and asserted that this ‘model prioritizes abstinence, being faithful to a monogamous partner, and only as a last result, condoms’ (Leventhal 2010, p. 204). Schlafly noted that the Catholic Church cares for one in four people treated for AIDS worldwide and that FBOs should play a key role in PEPFAR HIV prevention. PEPFAR legislation reflects similar principles:

… Uganda's successful AIDS treatment and prevention programme is referred to as the ABC model: “Abstain, Be faithful, use Condoms”, in order of priority. … Beginning in 1986, Uganda brought about a fundamental change in sexual behaviour by developing a low-cost programme with the message: “Stop having multiple partners. Be faithful. Teenagers, wait until you are married before you begin sex”. (PEPFAR I. H.R. 1298, p. 4)

Historically, Christian and Islamic proselytisers had gone to Africa earlier on and their indelible imprint remains. As Matua (2002) points out:

… At the core of attempts to subjugate Africans to the messianic tradition is a belief not only in the superiority of the missionary and his or her messianic dogma but also in the sub-humanity of the missionary's subjects and their cosmology. (p. 227)

Catholicism, Protestantism, Pentecostalism and Islamism have displaced many African traditional religious rituals (Mbiti 1969, Matua 2002). Similarly, PEPFAR and its conservative messages concerning HIV sexual-transmission prevention have been transported to Africa, with hopes of changing deep-rooted practices.

With the USA providing such a colossal sum of money through PEPFAR, which remains unparalleled in history, it is not surprising that US policy-makers would attempt to direct health care policy in PEPFAR recipient countries, including Nigeria. The PEPFAR Prevention Technical Working Group provided significant input into the revised 2003 Nigerian National Strategic Framework (PEPFAR 2008). The policy document employs similar strategies that are prominent features of PEPFAR legislative mandates. Some of these strategies do not necessarily reflect evidence-based measures or general consensus among public health practitioners. Rather, they aim to appease religious organisations and donor partners.

PEPFAR legislation charged the US Global AIDS Coordinator with establishing HIV sexual-prevention strategies governing the expenditure of PEPFAR funds in countries with generalised epidemics. The legislation earmarked funds for disbursement beginning in 2006–2008 as follows: 55% for HIV/AIDS treatment, 15% for palliative care for HIV victims, 20% for HIV/AIDS prevention programmes with at least 33% of this amount specifically for abstinence-before-marriage programmes and 10% to help orphans and other vulnerable children. Ingram (2007) cites the Center for Health and Gender Equity in stating that ‘one analysis estimates the share of funds channeled to “abstinence-only” strategies for the prevention of sexual transmission of HIV in Nigeria at 70%, far in excess of that implied by the legislative ceiling of 33% of prevention funds’ (p. 527). Ingram further notes PEPFAR's direct provision of antiretroviral drugs for Nigeria's military forces (commonly provided by the sovereign state) as a novel biopolitical commitment to protecting and maintaining a foreign military.

Generally, there is a marginal difference between PEPFAR practices in different countries in the region. The conservative religious undertone of PEPFAR is also apparent in other PEPFAR partner countries. The ABC model originated in Africa, and was borrowed and reshaped by the Bush Administration. The United States Government Accounting Office-06-395 (2006) notes that half of the country teams stated that ‘the spending requirement can undermine the integration of prevention programmes by forcing them to isolate funding for AB activities’ (p. 6). In 2007, prior to PEPFAR II (the Reauthorization Act), the US Institute of Medicine of the National Academies found that PEPFAR I budget allocations that set percentage levels on prevention, care, treatment and activities within prevention programmes (1) have adversely affected implementation programmes; (2) inhibited comprehensive, integrated and evidence-based approaches; (3) have been counterproductive; and (4) have limited the ability of PEPFAR programmes to tailor activities to meet country-specific epidemics and coordinated activities in countries’ national programmes. The US Senate had hearings on the lack of evidence-based approaches within PEPFAR (H.R. 5501, 2008). As Leventhal (2010) explains, senators promised to amend the legislation to better reflect local epidemiological needs. Thus, the Reauthorization Act calls for a balanced approach in prevention activities for sexual transmission, ‘… ensuring that activities promoting abstinence, delayed sexual debut, monogamy, fidelity, and partner reduction are implemented and funded in a meaningful and equitable way in the strategy for each host country based on objective epidemiological evidence’ (pp. 49–50). However, PEPFAR II specifies that the US Global AIDS Coordinator must report to the US Congress within 30 days if less than 50% of prevention funding is not spent on AB activities, a reiteration that PEPFAR would not radically deviate from its founding principles.

## Religion, morality and sexuality in HIV prevention programmes in Nigeria

In Nigeria, religious conservatives in many ways align with PEPFAR creators. There have also been concerted efforts between Nigerian and American conservatives to influence policies affecting people living with HIV/AIDS (PLWHA) in Nigeria. Hybrid alliances have been forged between these groups and their shared beliefs have led them to gravitate toward a common pole. In 2004, more than 30 FBOs and NGOs in Nigeria collaborated to create the Nigeria Abstinence Coalition. The organisation, an affiliate of the US-based Abstinence Clearinghouse, purportedly aims to advance abstinence and fidelity messages and practices (Abstinence Africa 2004, Human Rights Watch 2004).

Mafeni and Fajemisin (2003) note that during the early stages of the HIV epidemic, many churches in Nigeria adopted compulsory HIV testing before marriage, and refused to allow those found positive to get married in churches. Although this decision was later recanted by the highest levels of church leadership, the practice remained prevalent in local parishes. Local religious leaders may not fully comply with official positions of higher levels of leadership. Further, many evangelical churches are non-denominational and do not conform to a hierarchical structure. A majority of religious leaders in Nigeria share similar positions on premarital sex. Core religious messages, as suggested by Ucheaga and Hartwig (2010), emphasise abstinence outside of marriage and faithfulness within marriage. They note that fear messages are promoted among Christians and Muslims, and both groups threaten damnation and a life in hell for those who do not conform. Further, the Catholic Church vehemently opposes the use of condoms, asserting that it counters God's plans regarding procreation. Evertz (2010) notes that conservatives in the USA were less motivated to provide condoms to the world's population because it would appear to be an implicit endorsement of premarital sex.

Ucheaga and Hartwig (2010) note that the majority of religious leaders in Nigeria do not promote condom use for HIV prevention, and these leaders believe that collaboration with the Nigerian government is worthwhile as long as the government does not attempt to force religious organisations to support condom use. The general view is that condom use promotes promiscuity and is not a reliable means of HIV prevention. Notable exceptions to this view are the Anglican Church in Nigeria, some branches of the Lutheran Church and the All African Conference of Churches, which support the use of condoms (Iliffe 2006).

The conservative message regarding condom usage resonates not only with religious leaders, but also with some governmental and regulatory authorities in Nigeria. In 2001, a radio advertisement was suspended by the Advertising Practitioners Council of Nigeria because it was deemed to convey the message that premarital sex is acceptable as long as a condom is used (AVERT 2011). In 2006, stricter regulations on condom advertising were enforced so as to discourage presumed indecency. Aversions to condom use, although mainly framed in religious terms, have diverse antecedents. Iliffe (2006) attributes low condom use at the beginning of the HIV/AIDS epidemic in Africa to a widespread distaste among both men and women. Condoms were at first almost exclusively used among sex workers, such that women who insisted on condoms were viewed as prostitutes. Iliffe cites Mwangi's analysis that ‘the men were too manly to use them and the women were too womanly to insist’ (p. 134). Engel (2006) notes that many Africans associated condom use with efforts at genocide, homosexuality and emasculation. Some men rejected condom use because of the financial cost and others because they preferred sexual contact with flesh. Despite social aversions, the scientific community clearly views condom use as imperative in curbing the spread of the virus, while conservatives in the USA and Nigeria stubbornly propagate the contrary.

In 2004, the Nigerian president asserted that ‘homosexuality is clearly un-Biblical, un-natural, and definitely un-African’ (AVERT 2011). Homosexual practices are illegal and highly stigmatised in Nigeria. American conservative Christians have actively advocated for legislation criminalising homosexuality in some African countries. As an example, Uganda's Anti-Homosexuality Bill, supported by evangelical pastor Rick Warren, has been used as a model for the proposition of similar legislation in Nigeria.

In West Africa, according to Ucheaga and Hartwig (2010), people in organised religion primarily receive information on health-related behaviour, sexuality and morality from religious leaders. These leaders and their core messages are highly relevant in regard to how people perceive of themselves and HIV since the virus has been framed in terms of health, sexuality and morality. Dilger *et al.* (2011) observe that:

religious traditions are manifested in sexuality and reproduction; the emergence of HIV has been co-productive in the emergence of new religiosities that inform individual and social identities, and which consequently have a bearing on policies and political and economic realities. (p. 373)

PEPFAR legislation asserts that since many Africans are affiliated with a religious institution, FBOs possess important experience that is needed to carry out prevention programmes. PEPFAR Nigeria particularly encourages local FBOs to apply for PEPFAR funding. PEPFAR mobilises religious leaders to include HIV prevention messages in their weekly sermons in churches and mosques (PEPFAR 2008). These sermons reinforce messages of moral-sexual chastity in HIV/AIDS prevention.

Moral judgments are proselytised through religious beliefs, which in turn impact sexual practices promoted by AIDS prevention programmes. Dilger *et al*. (2011) further observe that in sub-Saharan Africa, certain Christian organisations have constructed HIV infection as a punishment from God, a belief that has resulted in the dichotomy between ‘good/pure’ and ‘bad/impure’ Christians. HIV/AIDS has been presented in the public discourse as divine retribution for individual or societal failure to adhere to sanctioned modes of conduct. Similarly, Smith (2004) notes that in Nigeria, the framing of HIV/AIDS as a social problem resulting from immorality reflects a common perception that the nation's most entrenched social problems also result from moral decadence. The framing of HIV/AIDS as divine retribution has been criticised by researchers and activists as contributing to the stigmatisation of PLWHA, which impedes efforts to reach those most at risk. Smith (2003) writes that ‘one prevailing discourse is that AIDS is a scourge visited by God on a society that has turned its back on religion and morality’ (p. 364), a belief that resonated with US conservatives at the onset of the HIV epidemic. Smith further suggests that Nigerian youth have a deterministic view regarding HIV infection. He notes that ‘strong religious ideas about the role of God in determining fate combined with a belief that God protects those who behave morally exacerbate a cycle of stigma and denial that very much characterises popular responses to AIDS in the communities’ (p. 346). People believe that God causes AIDS, and whether or not they become infected is determined by God (Smith).

Religious beliefs and morality extend beyond the realm of HIV infectivity. Prevention methods are also characterised in a dichotomy of godly or ungodly approaches. Balogun (2010) argues that in Nigeria, mainstream Muslims involved in policy-making regarding HIV/AIDS still appear to adhere to the view that campaigns for condom use tend to encourage unlawful (under Islamic law) sexual intercourse, which is unacceptable from a purist Islamic understanding. Thus, Balogun insists that most Muslims in Nigeria believe that solutions to curbing the HIV epidemic lie in Islamic teaching on sexual abstinence outside of wedlock and being faithful to one wedded partner. He further notes that Islam is not only a religion, but also a way of life that regulates all aspects of life including social activities, health and well-being. Yusuf and Baba (2010) echo a similar sentiment, claiming that the free distribution and sale of condoms encourage adolescent sexual promiscuity and contradict traditional values of chastity and virginity among newly married couples in Nigeria. Because of the sensitivity surrounding sex education and the types of messages to convey in sex education, prevention messages are inadequate. In 2009, only 22.8% of Nigerian schools had provided life skills-based HIV education during the past academic year, 11.7% of women and men ages 15–49 had received an HIV test in the past year and knew the results and 25% of men and women between the ages of 15 and 24 correctly identified ways to prevent the sexual transmission of HIV and rejected major misconceptions about HIV transmission (United Nations General Assembly Special Session 2010).

## Effects of PEPFAR activities in Nigeria

Despite these shortcomings, the positive effects of the biopolitical regime of PEPFAR are evident in Nigeria. As Foucault notes: ‘[It is] not that everything is bad, but that everything is dangerous, which is not exactly the same as bad’ (quoted in Elbe 2005, p. 13). One cannot ignore the fact that PEPFAR has worked to save lives in Nigeria, especially in areas such as mother-to-child HIV prevention programmes, safe blood supplies, care for orphans living with HIV and HIV treatment programmes. Other PEPFAR prevention activities include HIV counselling and testing services for pregnant women receiving antenatal care; encouraging HIV-positive pregnant women to disclose their status to partners and close relatives; providing antiretroviral treatment to positive pregnant women for prevention of mother-to-child HIV transmission (PMTCT); education regarding feeding options as prevention; early diagnosis and treatment of sexually transmitted infections to reduce HIV transmission; and prevention of medical HIV transmission (PEPFAR 2008). PEPFAR Nigeria engages in HIV awareness activities with the production of television and radio jingles, pamphlets and brochures. It elicits the help of a broad spectrum of community members, including teachers, factory managers and workers, parents and guardians, women, youth and men's groups and traditional and community leaders. PEPFAR Nigeria continues to train traditional birth attendants in PMTCT and to work toward increasing deliveries in medical facilities. Through PEPFAR funding, religious organisations have provided hospital care, counselling, mental health support and more health care professionals.

From 2004 to 2010, PEPFAR provided more than US$2.5 billion dollars to fight HIV/AIDS in Nigeria (PEPFAR 2012). More than 39 million Nigerians have been informed of prevention methods through community programmes; approximately 5 million Nigerians have been reached with HIV counselling and testing services; more than 2 million pregnant women have received PMTCT health services; over 1 million PLWHA have received care to support their quality of life; over 200,000 children orphaned by HIV/AIDS and other vulnerable children receive care; and approximately 340,000 women and children are receiving antiretroviral treatment. PMTCT activities have been expanded and decentralised in Nigeria. There are more PMTCT outlets in Nigeria currently, with PEPFAR supporting 651 sites as of September 2009 (PEPFAR 2008). In 2010, Nigeria was among the few countries prioritised by PEPFAR for supplementary (plus-up) funding to scale up services. This biopolitical intervention has helped to shape Nigeria's demographic indicators. However controversial some of its modi operandi, it must be acknowledged that through PEPFAR, life expectancy for many Nigerians has increased as HIV/AIDS stabilises. PEPFAR funding has ensured that many babies in Nigeria are born free from the menace of HIV and have the chance of surviving to adulthood. These lifesaving activities are commendable. It must also be recognised that with such large resources, PEPFAR has the potential to have a greater impact on the lives of more Nigerians; policies need to be more evidence-based and tailored to address systemic issues within the country's weak health care system.

The Bush Administration's preference for FBOs can be partially explained by local dynamics and also by structural and political constraints in Nigeria and other African bureaucracies. An important premise of PEPFAR practices has been the distrust of governmental sectors and reliance on non-governmental institutions (Pfeiffer *et al.* 2008; Pereira 2009). The widespread corruption and inefficiencies of many governments in the region gives some credence to the active recruitment of FBOs and other humanitarian organisations. Transparency International (2012) ranks Nigeria 139 out of 176 nations on its Corruption Perceptions Index. Notwithstanding, the shift from the biomedical model of HIV/AIDS prevention is problematic and demands redress. PEPFAR's emphasis on the role of FBOs and local religious authorities in implementing PEPFAR projects, despite many of these organisations’ records of exclusion and non-evidence-based practices, gives an impetus to reinforce messages such as the emanation of HIV from ungodly behaviour and the rejection of proven methods of curbing the disease.

US Republican Representative Chris Smith amended PEPFAR legislation to also include a conscience clause stating that FBOs have the right to deny or refuse services based on their religious beliefs. This means that those seeking health services, including HIV-related care, may be denied services. This arbitrary decision-making power on the part of health providers that receive PEPFAR funding sets a dangerous precedent. Any health care provider with differing personal beliefs, especially in nontransparent settings, may prevent a person from receiving much-needed lifesaving care.

Despite significant resources provided by PEPFAR in Nigeria, results indicating the level of HIV awareness among the population are mixed. Many Nigerians still do not have adequate information about HIV/AIDS, and condom use has not reached its coverage potential. Myths regarding HIV endure. By and large, the social construct of HIV in Nigeria negates the biomedical aspect of how the virus is transmitted. The emphasis of blood to blood or body-fluid to body-fluid transmission of HIV has shifted to matters of morality and sexuality. In the USA, the transmission of the virus was also framed in terms of sexuality and place of origin when it was thought to emanate from the four Hs of high risk: homosexual, heroin user, haemophiliac and Haitian (Gallo 2006). The epidemiology of the disease was distorted, and those considered part of high-risk groups were singled out for public excoriation. Victims were accused of engaging in sodomy, a term that reinforces a strong moral construct of the disease. Two decades later, PEPFAR emerged with new social constructs of HIV and the dichotomies of populations into high- and low-risk groups.

The convergence of religious conservative ideology concerning morality and HIV infectivity between American and Nigerian conservatives produces a fertile ground for the influence and expansion of PEPFAR activities in Nigeria. Religious leaders in Nigeria now possess not only spiritual assets, but also have material resources with the potential to separate and create room for stigmatisation and fear. Victims may choose not to know their HIV status, partly fearing the social repercussions. In a biopolitical paradigm, as noted by Foucault (1998), the institution of power is present at every level of the social body and is utilised through diverse institutions. Foucault argues that technologies of power employed tend to segregate and form social hierarchies, which guarantee relations of domination and effects of hegemony. A potential concern of biopolitical rationality is that in its attempt to protect population life, governmental rationality may resort to means that are detrimental to those already afflicted by the disease. Elbe (2009) notes that these practices ‘reside not in the prospect of excessive state mobilisation but rather in the international expansion and intensification of a set of practices that seek to structure the bodies and behaviors of individuals in conformity with a wide range of norms’ (p. 18). Elbe (2005) cautions that biopolitical strategies historically have been Janus-faced: on one hand, they had provided hospitals and universal health care systems; on the other end, they had provided the platform to engage in eugenics and mass death. Foucault (1998) further notes that sexuality becomes a theme of ‘political operations, economic interventions and ideological campaigns for raising standards of morality and responsibility’ (p. 146). He observes that ‘precocious sexuality was presented from the eighteenth century to the end of the nineteenth as an epidemic menace that risked compromising not only the future health of adults but the future of the entire society and species’ (pp. 146–147). Similar disciplinary practices are advanced in the contemporary era to normalise a set of behaviours. These disciplinary tactics emanate from both within and without in an effort to maintain life. The conservative ideology reflected in PEPFAR competed with the evidence-based and holistic approach that emphasised prevention, which includes condom use, destigmatisation and protection of the rights of PLWHA. As head of the WHO's Global AIDS Program, Jonathan Mann played a crucial role in destigmatising PLWHA. Mann insisted that at times of greatest unease, discrimination and exclusion were not the way forward in curbing the spread of HIV/AIDS. Instead, Mann argued that the human rights of PLWHA are not contrary to, but support public health rationalities (Iliffe 2006).

## Conclusion

Nigeria, a fragile democratic state that is emerging from political instability with scarce resources in terms of human development, technological advances and poor health systems, has not been able to muster sufficient resources to address the HIV/AIDS crisis. PEPFAR fills this void. The politics in the formation of PEPFAR was immensely ideologically driven, reflecting the biopolitical perspective of US conservatives: restoring health, fostering life, increasing longevity and decreasing mortality and moral imperatives regarding sexuality. Nigerian religious authorities and FBOs adhere to similar views and in some instances, formed alliances with their American counterparts. The efficacy of HIV prevention programmes in Nigeria has been suboptimal, as HIV infectivity and prevention are viewed through a dichotomy of godly and ungodly.

Liberal thinkers and advocacy groups were vocal during the Bush Administration regarding these practices and emphasised the need to remove ideologically driven conservative policies from PEPFAR programmes. Most of these policies are embedded in structural policy documents of partner countries. Nigeria's approach to HIV prevention highlights similar themes and would require a significant overhaul to improve outcomes. With the Obama Administration assuming the PEPFAR reins in 2009, much of PEPFAR policy has changed and is poised for future changes. The Obama Administration acknowledged the solid foundation built by the Bush Administration in addressing the global HIV/AIDS pandemic. In addition, the Obama Administration announced the Global Health Initiatives (GHI), which allocates US$51 billion dollars toward PEPFAR Reauthorization and US$12 billion dollars to other global health issues. As the current administration attempts to shift to a more holistic approach, ABC appears to no longer be the insignia of PEPFAR HIV prevention (Clinton 2010, Kamath and Jense 2010). Secretary of State Hillary Clinton (2010) asserts that the GHI:

represents a new approach, informed by new thinking and aimed at a new goal: To save the greatest possible number of lives, both by increasing our existing health programmes and by building upon them to help countries develop their own capacity to improve the health of their own people. (p. 3)

Clinton highlights that the USA is ‘moving beyond A-B-C-abstinence, be faithful, and consistent and correct use of condoms–to an A to Z approach to prevention’ (p. 6) as the US integrates medical, behavioural and structural interventions. Clinton (2010) states that the USA is shifting the focus from solving problems ‘… one at a time, to saving people, by considering more fully the circumstances of their lives and ensuring they can get the care they need most over the course of their lifetimes’ (p. 4). She also notes that the USA needs ‘… to lay the groundwork now for more progress down the road by tackling some of those systemic problems, and working with our partner countries to uproot the most deep-seated obstacles that impede their own people's health’ (p. 7).

As Obama begins his second term, the direction of PEPFAR is expected to continue on a more progressive trajectory that employs more holistic approaches to optimise outcomes. In order to do away with prescriptive prevention programmes that highlight moral imperatives and de-emphasise epidemiological specificity, the Obama Administration must work to ensure that embedded ideologies in countries’ strategic HIV prevention frameworks are replaced.

## References

[R1] Abstinence Africa (2004). Nigeria abstinence coalition formed.

[R2] AVERT (2011). HIV and AIDS in Nigeria.

[R3] Balogun A. (2010). Islamic perspectives on HIV/AIDS and antiretroviral treatment: the case of Nigeria. African journal of AIDS research.

[R4] Bassett T., Straus S. (2011). Defending democracy in Cộte d'lvoire. Foreign Affairs..

[R5] Carson J. (2010). The US embassy cables: the documents. The Guardian.

[R6] Carson J. (2011). Speech given at the Woodrow Wilson Center, Washington, DC (April 5, 2011) [online].

[R7] Clinton H. (2010). The global health initiative: the next phase of American leadership in health around the world.

[R8] Curtis B. (2002). Foucault on governmentality and population: the impossible discovery.

[R9] Canadian journal of sociology.

[R10] De Waal A. (2006). AIDS and power: why there is no political crisis—yet.

[R11] Dietrich J. (2007). The politics of PEPFAR: The President's Emergency Plan for AIDS Relief. Ethics and international affairs.

[R12] Dilger H., Burchardt M., van Dijk R. (2011). Introduction – the redemptive moment: HIV treatments and the production of new religious spaces. African journal of AIDS research.

[R13] Elbe S. (2005). AIDS, security and biopolitics. International relations.

[R14] Elbe S. (2009). Virus alert: security, governmentality and the AIDS pandemic.

[R15] Engel J. (2006). The epidemic: a global history of AIDS.

[R16] Evertz S. (2010). How ideology trumped science: why PEPFAR has failed to meet its potential.

[R17] Foucault M. (1998). The history of sexuality. vol. 1: the will to knowledge.

[R18] Gallo R (2006). A reflection on HIV/AIDS research after 25 years. Retrovirology.

[R19] Human Rights Watch (2004). Access to condoms and HIV/AIDS information: a global health and human rights concern.

[R20] Iliffe J. (2006). The African AIDS epidemic: a history.

[R21] Ingram A. (2005). Global leadership and global health: contending meta-narratives, divergent responses, fatal consequences. International relations.

[R22] Ingram A. (2007). HIV/AIDS, security and the geopolitics of US-Nigerian relations. Review of international political economy.

[R23] Kamath A., Jense R. (2010). Health systems strengthening mechanism for the Global Health Initiative. Journal of the American medical association.

[R24] Leventhal I. (2010). PEPFAR: preaching abstinence at the cost of global health and other misguided relief policies. Temple international and comparative law journal.

[R25] Mafeni J., Fajemisin O. (2003). HIV/AIDS in Nigeria: situation, response and prospects.

[R26] Matua M., Gearon L. (2002). Returning to My Roots: African “Religions” and the State. Human rights and religion: a reader.

[R27] Mbembe A. (2003). Necropolitics. Public culture.

[R28] Mbiti J.S. (1969). African religions and philosophy.

[R29] Nigerian National Strategic Framework (2010). National HIV/AIDS Strategic Framework 2010–2015.

[R30] PEPFAR (2008). FY2008 Country profile: Nigeria (Nigeria COP). Annual report to Congress.

[R31] PEPFAR (2011). PEPFAR achive.

[R32] PEPFAR (2012). Partnership to fight HIV/AIDS in Nigeria.

[R33] PEPFAR H. R. (1298). United States Leadership against HIV/AIDS, tuberculosis, and Malaria Act of 2003. 108th Congress of the United States of America [online].

[R34] PEPFAR H.R. (5501). Tom Lantos and Henry J. Hyde United States Global Leadership Against HIV/AIDS, Tuberculosis, and Malaria Reauthorization Act of 2008.

[R35] Pereira R., Bartelink B., Pape U. (2009). PEPFAR project implementation and the pursuit of the United States national interest. Political, social and religious dimensions in the fight against HIV/AIDS: negotiating worldviews, facing practical challenges.

[R36] Pereira R. (2011). Project, implementation, subversion: grasping the cycle of global liberal HIV/AIDS policy.

[R37] Pfeiffer J., Johnson W., Fort M., Shakow A., Hagopian A., Gloyd S., Gimbel-Sherr K. (2008). Strengthening health systems in poor countries: a code of conduct for nongovernmental organizations. American journal of public health.

[R38] Schlafly P. (2003). Letter to President Bush, April 16, 2003. Eagle Forum.

[R39] Smith D. (2003). Imagining HIV/AIDS: morality and perceptions of personal risk in Nigeria. Medical anthropology.

[R40] Smith D. (2004). Youth, sin and sex in Nigeria: Christianity and HIV/AIDS related beliefs and behaviour among rural-urban migrants. Culture, health and sexuality.

[R41] Transparency International (2012). Corruption perceptions index.

[R42] Ucheaga D., Hartwig K. (2010). Religious leaders’ response to AIDS in Nigeria. Global public health.

[R43] UNAIDS (2010). UNAIDS Report on the Global AIDS Epidemic.

[R44] United Nations General Assembly Special Session (UNGASS) (2010). UNGASS Country Progress Report: Nigeria.

[R45] United States Diplomatic Mission to Nigeria (2012). PEPFAR implementing partners.

[R46] United States Government Accounting Office-06-395 (2006). Global Health: Spending Requirement Presents Challenges for Allocating Prevention Funding Under the President's Emergency Plan for AIDS Relief.

[R47] Yusuf A., Baba I. (2010). Institutional responses and the people living with HIV/AIDS in Nigeria: the gaps in sustainable prevention, mitigation, care and support. European Journal of Social Sciences.

